# Apatinib-loaded CalliSpheres Beads for embolization in a rabbit VX2 liver tumor: characterization *in vitro*, pharmacokinetics and tumor response *in vivo*

**DOI:** 10.1080/10717544.2020.1818881

**Published:** 2020-09-14

**Authors:** Qin Shi, Yongning Lu, Songjiang Huang, Chen Zhou, Chongtu Yang, Jiacheng Liu, Jinqiang Ma, Bin Xiong

**Affiliations:** a Department of Radiology, Union Hospital, Tongji Medical College, Huazhong University of Science and Technology, Wuhan, China; b Department of Pharmacy, Union Hospital, Tongji Medical College, Huazhong University of Science and Technology, Wuhan, China; c Hubei Province Key Laboratory of Molecular Imaging, Wuhan, China

**Keywords:** Liver cancer, transarterial chemoembolization, CalliSpheres beads, apatinib, pharmacokinetics

## Abstract

Apatinib mesylate is an oral antiangiogenic agent that can inhibit activation of vascular endothelial growth factor receptor-2 tyrosine kinase. However, its therapeutic use in liver cancer is restricted due to severe systemic toxicity. Our work aimed to construct apatinib-loaded CalliSpheres Beads (CBAPA) and investigate its application in transarterial chemoembolization (TACE) of liver cancer. The established stock solution containing 20, 40 or 60 mg apatinib were fully mixed with 100–300 μm CalliSpheres Beads (CB) for 2 hours, respectively. The highest loading efficiency at 30 min after combination in 20 mg group (maximum 70.7%). Further, apatinib can be steadily released from CBAPA *in vitro* release test. For pharmacokinetics and tumor response *in vivo*, sixty New Zealand white rabbits with VX2 liver tumor were assigned into four groups: sham (NS) group, apatinib solution alone (APA) group, CB group and CBAPA group. Apatinib was measured in plasma and liver tissue by high performance liquid chromatography–tandem mass spectrometry. Compared to APA group, the administration of apatinib by TACE with CBAPA resulted in low systemic concentration. In addition, intratumoural apatinib concentration was higher than adjacent hepatic parenchyma in the CBAPA group. Compared to other three groups, CBAPA group achieved lower tumor growth rate and improved survival time. In conclusion, these findings provide a basis for the potential application of apatinib-loaded CalliSpheres Beads in liver cancer.

## Introduction

Transarterial chemoembolization (TACE) is one of the most widely used methods in intermediate-advanced stage hepatocellular carcinoma (HCC) (Forner et al., 2018). Chemotherapeutic agents like doxorubicin or epirubicin could be suspended in lipiodol or other embolic materials are infused through tumor-feeding artery and then followed by gelatin sponge particles (Shirono et al., 2019). Drug-eluting beads (DEB), which can be used as a carrier for various antineoplastic agents (especially doxorubicin) in TACE, is considered as an effective embolic material for HCC (Namur et al., 2010). It has more benefit in terms of ability of embolization and effectiveness of drug delivery system compared to conventional TACE with lipiodol (Raoul et al., 2019). However, it seems to be similar in the therapeutic effect for HCC between the two options according to the guidance (Omata et al., 2017). The reason may be that doxorubicin is not an ideal agent for embolization treatment (Boulin et al., 2011). In other words, a suitable alternative drug is crucial to improve therapeutic effect.

Tumor ischemia after embolization can contribute to the high expression of hypoxia inducing factor (HIF-1α), and then drives the activation of vascular endothelial growth factor (VEGF) (Xu et al., 2014; Liu et al., 2017). The combination of VEGF and its receptors (VEGFR) can stimulate downstream pathways of the cascade, and finally leads to tumor angiogenesis, recurrence and metastasis (Li et al., 2014; Viallard & Larrivée, 2017). Based on it, inhibition of VEGF pathway is considered as a potential approach to control tumor growth. Apatinib mesylate is an oral antiangiogenic agent that can inhibit activation of VEGFR-2 tyrosine kinase. It has been approved as a second-line therapy in Chinese patients with advanced HCC. The systemic adverse events including gastrointestinal reaction, hypertension, abnormal coagulation function, proteinuria after oral administration were still common and even serious (Zhao et al., 2018), although improved survival occurred in advanced HCC, non-small-cell lung cancer and colorectal cancer (Liao et al., 2018; Wang et al., 2018; Wu et al., 2018). The local delivery of drug is a novel method to increase tumor drug concentration and reduce systemic toxicity (Zhou et al., 2020). CalliSpheres Beads (CB), the first DEB product for embolization in China, has better efficiency and safety in TACE (Ma et al., 2019; Shi et al., 2020). Hence, the combination of angiogenesis inhibitor apatinib and embolic material CB may be a potential method for TACE of liver cancer.

The present study aimed to construct the apatinib-loaded CalliSpheres Beads (CBAPA), investigate its loadability and releasing profiles *in vitro* and further evaluate the pharmacokinetic features of apatinib and tumor response *in vivo*.

## Materials and methods

### Materials preparation

Apatinib (Hengrui Medicine Co. Ltd., Jiangsu, China) was reconstituted with normal saline containing 20% ethanol and hydrochloric acid in an equimolar quantity to obtain 5 mg/mL stock solution. CB with size of 100–300 μm (Hengrui Medicine Co. Ltd., Jiangsu, China) was used for the study.

### Characterization of apatinib-loaded CalliSpheres Beads

100–300 μm CB (1 g) was added into the stock solution containing 20, 40 and 60 mg of apatinib, respectively. The mixtures were kept for 120 minutes in a shaker at room temperature. An equal amount of supernatant was collected from mixtures at 10 min, 20 min, 30 min, 60 min, 120 min, respectively. The drug concentration was detected by using EnSpire^®^ Multimode Plate Reader (PerkinElmer Co., USA) at 345 nm. Apatinib loading efficiency at each time point was indirectly calculated. All experiments were performed in triplicate.

The *in vitro* release test was conducted according to the established centrifuge method. Briefly, CBAPA was suspended in 10 mL normal saline contain 1% Tween 80 and incubated at 37 °C on a flat shaker (TS-110 × 30, TENSUC, Shanghai, China) at 100 rpm. At predetermined time intervals, the aliquots were taken for analysis, and the same volume of blank medium was added. After centrifugation, the supernatant was analyzed by EnSpire^®^ Multimode Plate Reader as describe above, and apatinib releasing rate at each time point was calculated. All experiments were performed in triplicate.

### Study design and animal model

The current study was approved by the Institutional Animal Care and Use Committee. All animals were brought from the Experimental Animal Center, and could free access to adequate food and water. Sixty adult New Zealand white rabbits weighing 2.0–2.5 kg were assigned into four groups: sham (NS) group (treated with 0.4 mL normal saline; *n* = 10), apatinib solution alone (APA) group (treated with 0.4 mL apatinib solution; *n* = 15), the CB group (treated with 0.1 g blank CB; *n* = 10); the CBAPA group (treated with 0.4 mL mixture of CBAPA; *n* = 25).

The rabbit VX2 tumor cells were bred in the muscle of the right thigh of a rabbit. The rabbit was sacrificed and tumor tissue was removed from the muscle when the tumor grew to a palpable mass. The tumor tissue was cut and separated into cubes at the size of 1 mm^3^ under sterile conditions and then these pieces were stored in saline. Next, incision was made in rabbits along the abdominal white line after anesthetized. Under aseptic conditions, a tumor piece was embedded into the hepatic left lateral lobe of each rabbit and then blocked with gelatin sponge, in order to prevent the tumor mass from falling out or liver bleeding. Finally, the wound was sutured after ensuring no bleeding or complications. All rabbits were given intramuscular injection of penicillin for 3 days after the implantation of tumor piece. The liver tumor growth situation was evaluated by a spiral contrast-enhanced computed tomography (CT; SOMATOM Sensation 64 Spiral, Siemens, Munich, Germany) after 15 days.

### Transarterial chemoembolization procedure

Hepatic arteriography was performed in all groups of tumor-bearing rabbits on digital subtraction arteriography (DSA; Siemens Medical Solutions, Munich, Germany). All animals were anesthetized before treatment, the preparation and sterilization of skin, an incision of skin and muscle, and separation of the femoral artery were performed in sequence. The 4-F sheath was then placed in the separated artery by a superb puncture technique. After that, a 4-F Cobra catheter (Cook, Inc., Bloomington, IN) was usually used to select the celiac artery for angiography to determine the hepatic artery. Next, a 2.7-F coaxial microcatheter system (Terumo, Tokyo, Japan) was further to select the hepatic artery and tumor-feeding artery (generally the left branch of hepatic artery) for angiography. When it confirmed that the complete feeding artery of tumor without other vessels, the treatment methods were performed in the different groups, and angiography was reviewed after treatment. At last, all catheters and sheaths removed, the wound was sutured. The penicillin was injected for 3 days in all rabbits as describe above.

### Sample collection

The plasmatic samples were collected though marginal ear vessels of rabbits at 5 min, 10 min, 30 min, 1 h, 3 h, and 6 h after treatment for CBAPA group and APA group. Then serum was isolated and stored at −80 °C until apatinib analysis.

Liver samples were collected at 6 hours, 3 days, and 7 days after treatment for CBAPA group. The VX2 tumor and adjacent hepatic parenchyma were harvested. Two kinds of tissue samples were weighted and homogenized by adding 0.9% saline solutions (10 mL/g sample weight), respectively. After centrifugation, the supernatant was frozen at −80 °C until apatinib analysis.

### Measurement of apatinib concentration in plasma and liver tissues

According to the pharmacopeia, apatinib concentration was analyzed by high performance liquid chromatography–tandem mass spectrometry (HPLC-MS/MS) with API 4000 triple quadrupole instrument (Applied Biosystems, USA). The standard curve in plasma ranging from 5 to 1000 ng/mL was established by mixed blank plasma samples (10 μL) with apatinib standard solution (50 μL). Ponatinib (Meilun Biotechnology Co., Dalian, China) at a concentration of 100 ng/mL and dose of 10 μL, as internal standard solution, were added in each plasmatic sample and standard solution. The plasmatic samples were pretreated with protein precipitation by adding 140 μL of methanol. Utimate XB-18 chromatographic column (2.1 × 100 mm,5 μm; Welch Materials, China) was used, and the flow rate was set as 0.3 mL/min. The injection volume of standards or samples was 5 μL. Measurement of apatinib concentration was performed on HPLC-MS/MS system. The lowest limit of quantity for apatinib was set as 5 ng/mL.

Apatinib concentration in tumor and adjacent hepatic parenchyma was analyzed by using the similar method as plasmatic samples. The standard curve in beforehand homogenates ranging from 5 to 5000 ng/mL was established. And lowest limit of quantity of HPLC-MS/MS for apatinib was set as above. The conversion of unit from ng/mL to ng/g was performed by multiplying the corresponding value to represent the apatinib concentration in tissue.

### Assessment of tumor response

The tumor size changes before and 7 days after treatment were monitored with contrast-enhanced CT. After that, we processed the imaging data by the Syngo Fastview image processing system. And tumor size, tumor growth rate and metastasis of the implanted VX2 liver tumor were assessed according to the modified Response Evaluation Criteria in Solid Tumors (mRECIST) criteria (Lencioni & Llovet, 2010). Tumor volume was calculated by formula: V = a x b^2^/2 (a, long diameter; b, short diameter). And tumor growth rate was calculated by formula: V_7_/V_0_ * 100%.

Five animals were euthanized at 7 days after treatment in the four groups. The tumor samples were harvested and preserved in formalin fixative solution. Tissue section was stained with hematoxylin-eosin (H&E, Sigma-Aldrich) to assess tumor necrosis, and immunohistochemical analysis of CD31 antibody (dilution: 1:100; DAKO, USA) was performed to assess tumor microvessel density. In addition, survival time after treatment was recorded in the four groups.

### Statistical analysis

All data were described as mean value ± standard deviation and processed by SPSS Statistics version 24.0 (SPSS Inc., Chicago, USA), and a *p* value < .05 indicated significant difference. The statistical methods of one-way ANOVA and LSD test were used to analyze the groups. Survival curves were determined by Kaplan-Meier methods. The figures were made by GraphPad Prism 8.00 software (GraphPad Software, La Jolla, USA).

## Results

### *Apatinib loading efficiency and release percentage* in vitro

**Figure 1.**
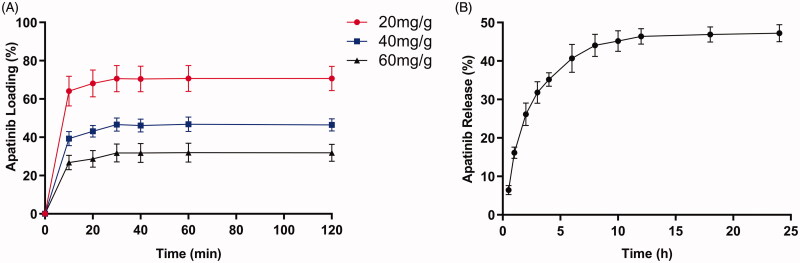
(A) Percentage curves of various apatinib loading. (B) Percentage curve of apatinib release in 20 mg/g group.

Apatinib loading efficiency peaked at 30 min in 20 mg/g group, 40 mg/g group and 60 mg/g group (Figure 1(A)). The mean maximum loadability was 70.7%, 46.6% and 31.8% in 20 mg/g group, 40 mg/g group and 60 mg/g group, respectively. Besides, apatinib loading efficiency kept level after 30 min in each group. The result revealed 20 mg/g group can present highest apatinib loading efficiency. As to apatinib release profile, apatinib release percentage gradually increased within 12 h in 20 mg/g group and then kept stable (Figure 1(B)). The total drug release percentage reached 47.2% within 24 h.

### Apatinib concentration in plasma

**Figure 2.**
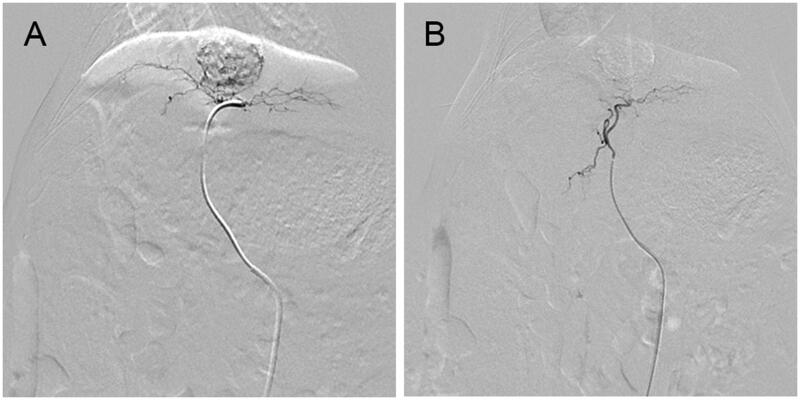
Arterial angiography of a rabbit VX2 liver tumor before treatment (A) and immediately after treatment (B) with CBAPA. The tumor staining disappeared after embolization.

**Figure 3.**
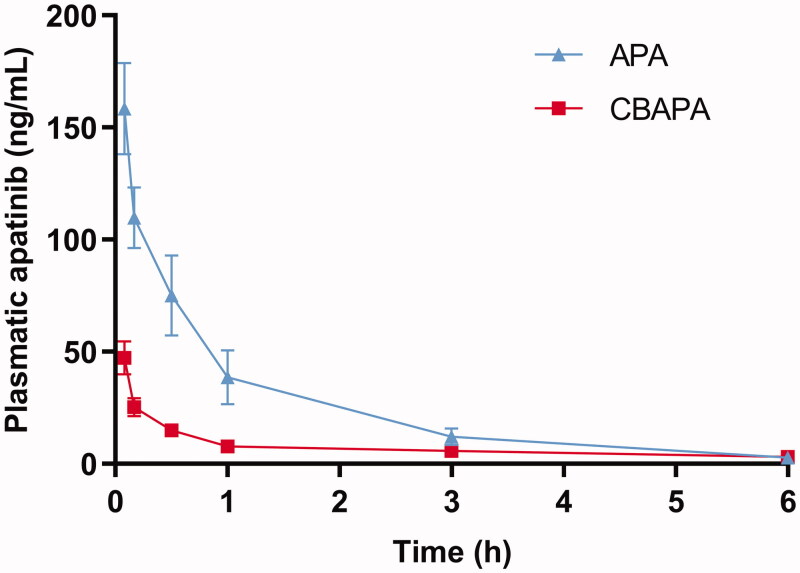
Plasmatic apatinib (ng/mL) curves of apatinib after treatment in the CBAPA and APA group.

TACE were performed successfully in all rabbits by the same operators (Figure 2). Besides, the plasmatic samples were detected and apatinib concentration decreased over time in the CBAPA group and APA group (Figure 3). Plasmatic apatinib concentration reached a maximum at 5 min after treatment for CBAPA group and APA group (47.26 ± 7.41 ng/mL vs 158.40 ± 20.43 ng/mL, *p* < .001). Meanwhile, at 10 min (25.22 ± 3.96 ng/mL vs 109.80 ± 13.61 ng/mL, *p* < .001), 30 min (14.88 ± 2.55 ng/mL vs 75.02 ± 17.92 ng/mL, *p* < .001), 1 h (7.74 ± 1.30 ng/mL vs 38.58 ± 11.95 ng/mL, *p* < .001) and 3 h (5.76 ± 0.39 ng/mL vs 10.62 ± 5.93 ng/mL, *p* < .05) after treatment, CBAPA group had obviously lower plasma apatinib concentration compared to APA group. It was similar at 6 h after treatment between the two groups (*p* > .05).

### Apatinib concentration in tumor and adjacent hepatic parenchyma

**Figure 4.**
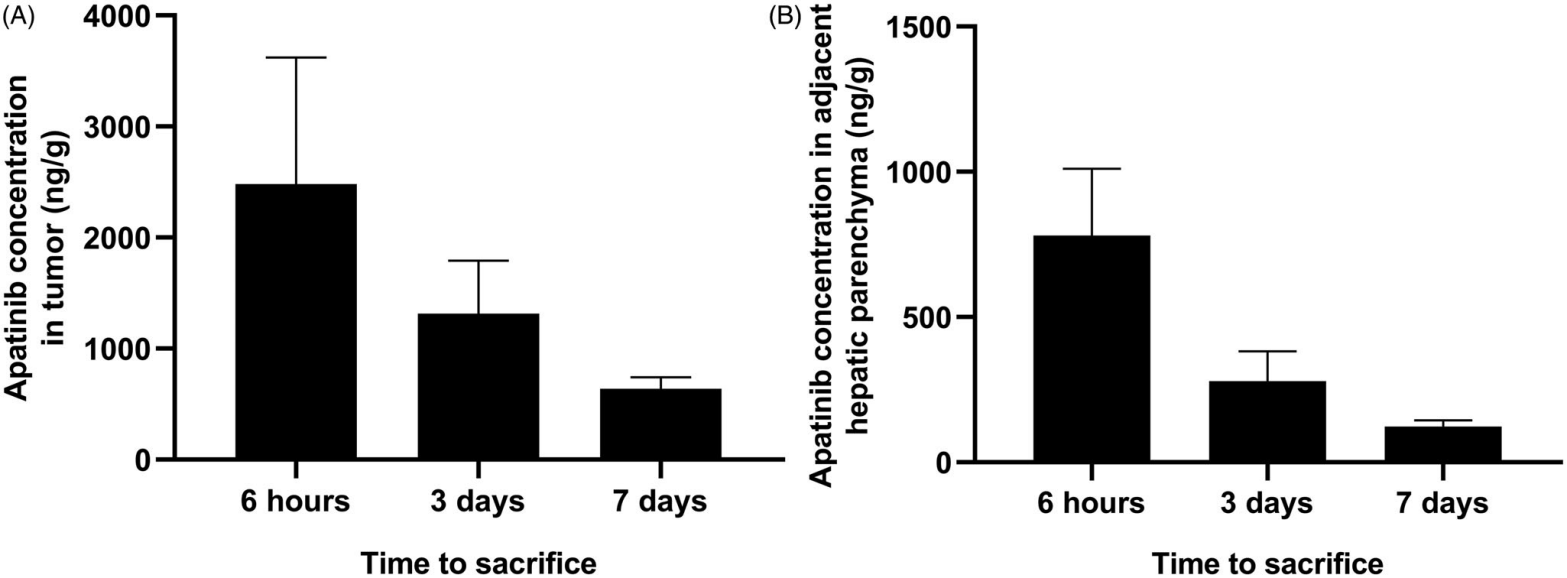
Apatinib concentration (ng/g) in tumor (A) and adjacent hepatic parenchyma (B) at 6 hours, 3 days and 7 days after TACE with CBAPA.

Rabbits with VX2 liver tumor were sacrificed and liver samples were collected at 6 hours, 3 days, 7 days after treatment for CBAPA group (Figure 4). The highest intratumoral apatinib concentration were observed at 6 hours after treatment (2480.7 ± 1140.9 ng/g), and then gradually reduced to 1316.1 ± 474.5 ng/g and 637.5 ± 104.6 ng/g at 3 days and 7 days after treatment, respectively. In the adjacent hepatic parenchyma, apatinib concentration was observed at 6 hours (780.2 ± 230.8 ng/g), 3 days (278.9 ± 102.8 ng/g) and 7 days (122.9 ± 21.0 ng/g) after treatment. Briefly, apatinib concentration in tumor was higher than adjacent hepatic parenchyma at each time point after TACE with CBAPA (*p* < .05).

### *Tumor response and survival* in vivo

**Figure 5.**
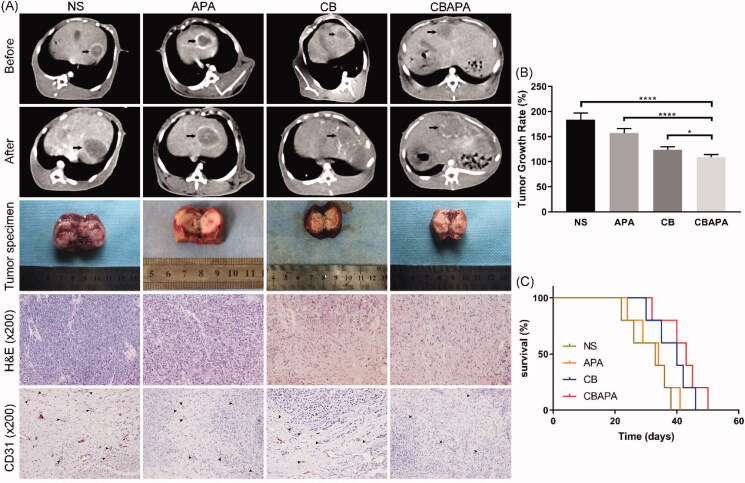
Tumor response *in vivo*. (A) Abdominal dynamic CT scan was performed on rabbits before and 7 days after treatment. The tumor specimen of each rabbit liver was taken 7 days after treatment. Further, histopathology of H&E staining and CD31 staining were performed on the specimens. (B) Tumor growth rate in the four groups. ****p < .0001, *p < .05. (C) Survival curves in the four groups.

Rabbits VX2 liver tumor changes before and 7 days after treatment were compared in four groups by imaging and histopathology (Figure 5(A)). Compared to NS and APA group, massive necrosis occurred in tumor area at 7 days after embolization for CBAPA and CB group in the dynamic CT scan. Subsequently, the tumor gross specimen was taken and further explained this result. H&E staining revealed fewer residual tumor cells were found in CBAPA and CB group compared to NS and APA group. Besides, CD31 staining was performed to observe the tumor microvessels density after treatment in the four groups. Compared to NS, APA and CB group, lower expression of microvessels density was detected in CBAPA group. This may reveal TACE with CBAPA can inhibit tumor angiogenesis. Importantly, tumor growth rate was calculated in the four groups according to the above formula (Figure 5(B)). The result indicated a lower tumor growth rate in CBAPA group than other three groups (*p* < .05). Besides, survival time was recorded and the mean value was 31.0 ± 6.8 days, 32.8 ± 6.5 days, 38.6 ± 6.2 days, 42.0 ± 6.7 days in NS group, APA group, CB group and CBAPA group, respectively (Figure 5(C)). A longer survival time occurred in CBAPA group than other three groups (*p* < .05).

## Discussion

According to the statistics, there are approximately 466.1 thousand new cases of HCC every year in China, accounting for almost 50% in the world, which implies a huge demand for TACE or targeted treatment (Chen et al., 2016; Zhu et al., 2016). DEB, as mentioned above, can achieve maximal tumor drug concentration with minimal systemic level (Varela et al., 2007). It is composed of a polyvinyl alcohol hybrid hydrogel linked to sulfonate groups, negatively charged of which provides for binding sites that allow drugs with positively charged for interaction (Lewis, 2009). In recent years, TACE with CB (a kind of DEB made in China) has been used for the treatment of hypervascular tumor (Peng et al., 2020). Besides, some studies indicated combination of TACE and oral antiangiogenic drugs can yield significant clinical benefit in the treatment of advanced HCC (Zhu et al., 2014; Liu et al., 2020). Unfortunately, some antiangiogenic drugs are restricted to some HCC patients due to severe systemic toxicity. To avoid it, a previous study revealed that DC Beads could be loaded efficiently with multi-targeted tyrosine kinase inhibitor sunitinib and would be a potential application for vascular embolization. However, the effort failed to precisely represent the pharmacokinetics of apatinib and tumor response in liver cancer (Fuchs et al., 2014). In the present study, we combined angiogenesis inhibitor apatinib with embolic material CB and evaluated its application in a rabbit VX2 liver tumor.

Unlike chemotherapeutic agents, most of angiogenesis inhibitors are insoluble in water by using conventional method. Sorafenib is the most common angiogenesis inhibitor for advanced HCC worldwide and its liquid solution formulation was prepared by using a solvent of 12.5% Cremophor EL, 12.5% ethanol and 75% distilled water in a previous study (Gaba et al., 2013). Besides, sunitinib (a multitargeted tyrosine kinase inhibitor) was dissolved in 5% glucose solution containing an appropriate acid in the Fuchs et al. study (2014). In the study, we explored the solubility of apatinib and dissolved it in normal saline containing 20% ethanol and an equimolar hydrochloric acid. *In vitro*, CB had a high loading efficiency of apatinib, and it failed to be improved significantly as drug solution increased. Apatinib amount was negatively correlated with loading efficiency, which may imply the limited number of sulfonate groups on the surface of the beads are not enough to bind more positively charged apatinib. Han et al. (2019) used CB to be loaded with oxaliplatin, which is a little similar trend to our result of loading efficiency. These data suggest CB has a good loadability.

To evaluate pharmacokinetic profile of apatinib and tumor response reasonably, we chose a relatively small dose (2 mg/per rabbit) because of two considerations. First, increasing evidences found low dose of antiangiogenic treatment was effective in improving the tumor microenvironment (Huang et al., 2013; Zhao et al., 2019). Second, substantial parenchymal damage was seen in rabbits with large dose of antiangiogenic treatment in the study by Seidensticker et al. (2016), while no significant liver damage was seen in rabbits with low dose in other study (Chatziioannou et al., 2013).

Pharmacokinetic profile of apatinib with oral administration has been evaluated in population in a study (Yu et al., 2017). However, the characterization of apatinib concentration in plasma and tumor after local administration is still unknown. In the current study, Plasmatic apatinib concentration presented a sustained downward trend within 1 h after treatment for CBAPA group and APA group. Subsequently, it tended to be stable in CBAPA group, while still declined in APA group. In addition, apatinib concentration was significantly higher in tumor than adjacent hepatic parenchyma after TACE with CBAPA. The highest intratumoral apatinib concentration were observed at 6 hours after CBAPA treatment and then gradually reduced. These findings further explain CBAPA can exhibit high intratumoral apatinib accumulation and low systemic exposure, which may provide a basis for reducing the occurrence of systemic adverse events.

Besides, a previous study proved an effective strategy for combining transcatheter arterial embolization with iodized oil containing apatinib to inhibit HCC growth and metastasis in a rabbit model (Zhou et al., 2020). The activity of RAF-MEK-ERK, PI3K/Akt, and P38 MAPK signaling pathways had been downgraded by apatinib. Importantly, it can help inhibiting tumor growth and angiogenesis in an anoxic microenvironment. In the present study, TACE with CBAPA had a great killing effect for tumor, which benefited from sustained apatinib releasing and antiangiogenic effect in tumor. Besides, the efficacy of in-situ apatinib administration alone was not ideal. The reason may be the frequency and doses were not enough to kill the tumor thoroughly.

There were several limitations in the study. Firstly, the comparison of CBAPA, apatinib with lipiodol and doxorubicin with CB had not been performed, which would be further confirmed in the future study. Secondly, the number of animals in each group were relatively small, which might reduce statistical efficiency.

In conclusion, angiogenesis inhibitor apatinib could be effectively combined with CB. TACE with CBAPA can achieve high intratumoral drug concentration and low systemic levels. In addition, the combination of CB and apatinib can inhibit tumor angiogenesis, slow tumor growth and improve survival time. These findings make it a promising potential therapy for the treatment of HCC.
